# Senior medical student perceived ability and experience in giving peer feedback in formative long case examinations

**DOI:** 10.1186/1472-6920-13-79

**Published:** 2013-05-31

**Authors:** Annette W Burgess, Chris Roberts, Kirsten I Black, Craig Mellis

**Affiliations:** 1 Sydney Medical School - Central, The University of Sydney, Sydney, New South Wales, Australia; 2 Sydney Medical School – Northern, The University of Sydney, Sydney, New South Wales, Australia

## Abstract

**Background:**

Learning to provide feedback on a peer’s performance in formative clinical assessments can be a valuable way of enriching the students’ own learning experience. Students are often reluctant to provide honest, critical feedback to their peers. Nevertheless, it is an area of practice that is important to develop as students report feeling ill prepared in feedback techniques when entering the medical workforce. We sought to investigate students’ perceptions of their ability to provide feedback to their peers using the positive critique method, and their perceived benefits and challenges during the experience.

**Methods:**

Over a two year period (2011 to 2012), senior medical students assessed and gave feedback to their peers alongside academic examiners during formative long case clinical examinations. Rating scales, open ended questions and focus group discussions were used to evaluate student perceptions.

**Results:**

Of the 94 participants, 89/94 (95%) completed the questionnaire, and 39/94 (41%) participated in focus groups. Students found the positive critique method provided a useful framework. Some students raised concerns about the accuracy of their feedback, and felt that further training was required. A substantial number of respondents (42%) did not report feeling confident providing negative feedback to their peers, and qualitative analysis indicated concerns around potential impacts on social relationships. Despite these concerns, the majority (90%) of respondents found the exercise useful, identifying several benefits, including development in the understanding of knowledge content; development of professionalism skills, and increased responsibility.

**Conclusion:**

Students identified several challenging aspects to providing feedback to their peers. While the experience of giving feedback to peers was perceived by students to provide a valuable learning experience, further training in this area may help to improve the learning experience for students and better prepare them for their future careers.

## Background

Providing feedback to peers within formative clinical examinations can provide a valuable method to enrich the students’ own learning experiences. Feedback within the context of clinical education has been defined as *“Specific information about the comparison between a trainee’s observed performance and a standard, given with the intent to improve the trainee’s performance”*[1]. By providing students with accurate feedback, the gap between actual and desired performance can be narrowed [2]. Students who provide feedback to their peers report metacognitive gains; increased student responsibility; and development of professionalism skills [3]. Although giving feedback is an essential component of a lifelong career in medicine, it is an area of practice where junior doctors often feel ill prepared on entering the workforce [4-6]. While university students show engagement in the experience and perceive many benefits, concerns regarding students’ reluctance to provide accurate, critical feedback to their peers are widely reported [3].

Appropriate student peer feedback may not occur for many reasons including social discomfort; associated responsibility; and inadequate training [7]. Providing constructive feedback that covers both positive and negative aspects of a performance is a difficult task. However, the negative effects of not giving feedback can be substantial, as good performance is not reinforced and a poor performance remains uncorrected [8]. Feedback can also cause harm if not carefully relayed, particularly if the feedback is negative, which may result in demotivation or deterioration in performance [8]. Several medical programs have sought to provide a structured learning situation in which feedback can be given, and received in a supportive manner. We report on a setting where students had the opportunity to provide feedback to their peers within a structured peer assessment exercise modelled on the traditional clinical long case.

It is not clear whether feedback from peers in such a setting is accurate and valuable [9]. Vickery & Lake (2005) state that good feedback requires clear goals and outcomes; direct observation of learners; and skills in giving both positive and negative feedback [5], and recommend following Pendelton’s positive critique method [10,11]. Within a context of peer assessment, the framework encourages self-reflection, and emphasises positive aspects of performance, rather than providing a method to avoid negative feedback. For example, when used correctly, this method can make delivery of feedback easier on the student assessor, as it allows the student being assessed to speak first regarding their needs for improvement. Rather than having the assessor raise these issues first, the assessor can concentrate on providing specific examples, and define areas for improvement.

### Context

The clinical school in which this study took place was a large tertiary teaching hospital, and one of six clinical schools to which students were allocated in the final two years of a four year graduate entry problem based medical program. As part of the assessment strategy, students were required to undertake a formative long case clinical examination. These formative examinations are designed to inform the students of their strengths and weaknesses in preparation for their summative examinations. Students were required to act as assessors of their peers, alongside an academic examiner.

In this context we specifically sought to investigate students’ perceptions of their ability to provide feedback to their peers, and their perceived benefits and challenges during the experience.

## Methods

The study was conducted over a two year period from 2011 to 2012, and involved Year 4 students.

Prior to the formative examinations, students were provided with a one hour training session facilitated by two of the authors. Here, the marking domains and marking criteria of the formative long case were explained, and students were given guidance on how to lead the questioning of their peers. Students were also provided with specific instructions on how to provide feedback to their colleagues using Pendelton’s positive critique method [12] which is summarised in Table 1.

**Table 1:** Pendelton’s positive critique method (Pendelton et al, 2003)

1.	Ask the student what went well
2.	Tell them what went well
3.	Ask the student what could be improved
4	Tell them what could be improved

The study was conducted using mixed methods to collect quantitative and qualitative data to assess student perception of their ability and experience of providing feedback to their peers.

### Quantitative data

Immediately following each long case examination, survey questionnaires were distributed to all student assessors, using both closed and open ended questions. The survey questions were based on Brookfield’s Critical Incident Questionnaire, which was designed to provide significant feedback on student experiences in the learning environment [13]. Students were asked to respond to closed ended items using a Likert-scale of one to five, with 1 being “strongly disagree”, 2 “disagree”, 3 “neutral”, 4 “agree” and 5 “strongly agree”. Descriptive statistics were used to analyse these data [14].

### Qualitative data

The questionnaire also included two open-ended questions aimed at eliciting responses from students regarding the “most useful” and “most difficult” aspects of delivering feedback to their peers. At the completion of each set of long cases examinations, all students were invited to attend focus groups. The focus group questions were designed to explore aspects of the students’ experience of giving feedback to their peers, in greater depth, for example, “What did you think about providing feedback to your peers?” and “Did providing feedback to peers help you in your own learning?”. Focus group data were transcribed verbatim with each participant being assigned an anonymous identifier (1 to 39). The combined data were read by the first author and analysed to identify themes. Following negotiation of meaning with the second and third authors, a coding framework was developed and applied to the full data set [14]. NVivo qualitative data analysis software was used for data analysis and management.

Ethics approval was obtained from The University of Sydney Ethics Committee.

## Results

**Figure 1:**
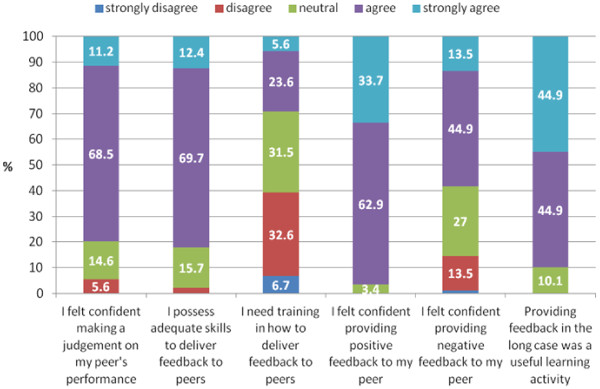
Stacked bar chart: student responses to questions using a 5 point Likert scale ranging from 1 being “strongly disagree”, 2 being “disagree”, 3 being “neutral”, 4 being “agree” and 5 being “strongly agree”.

Of the 94 participants, 89/94 (95%) completed the questionnaire. The responses are displayed in Figure 1. The main findings were that 80% of respondents reported feeling confident to make a judgement on a peer’s performance and 82% of respondents felt that they possessed adequate skills to deliver feedback. However, a significant number of respondents (42%) didn’t feel confident in providing negative feedback to their peers. Surprisingly 71% of respondents indicated that they did not need further training. However, most respondents (90%) found giving feedback to be a useful learning activity. These findings were unpacked to provide a richer understanding through the qualitative data.

Qualitative data consisted of open ended question responses (to which there was a response rate of 89/94, 95%), and a total of six focus groups, in which 41% (39/94) of students participated over the two year period. The qualitative findings contextualised the questionnaire results.

Analysis revealed three main themes. First, social discomfort in providing feedback to peers, including concerns regarding the accuracy of feedback, and the adequacy of their training. Second, the opportunity for self-reflection of clinical knowledge and skills, and third, the development of professionalism attributes including a sense of responsibility to assist their peers.

The most challenging aspect for students was being expected to provide negative feedback to their peers, with 33/89 (37%) of students commenting that they found it difficult to deliver negative feedback. They remarked that they found it “awkward” and “uncomfortable” and feared being overcritical.

I felt like I was being cruel, like how would I know better than them. I found it awkward (giving feedback) but making the judgement helped me learn a lot. S10

The social discomfort for students in giving feedback in our study resonated with the findings of Cassidy (2006) [7]. However, students noted that having a positive critique method [12] to follow was helpful to them.

“..if you frame it in a constructive way rather than just being critical, then it is always going to be okay.…you always ask them to reflect first and you go through the positives, and then after they have reflected, you can give your view as well. I think that’s a good way to do it… the way which it’s given is probably the most important thing.” S12

Students also had concerns regarding the accuracy of their feedback and felt discomfort with the responsibility they associated with their judgements. Seventeen percent (15/89) of respondents said that they did not feel competent enough to make a judgement on their peer’s performance, and could not be sure that the feedback they were giving was correct.

I feel like I’m not qualified to give feedback. S9

As noted by Cassidy (2006), a small but significant minority of students had concerns about the adequacy of the training provided [7]. Thirteen percent (12/89) of respondents commented that they were “unsure how to be constructive” in their feedback and felt that they needed “more training” (S15). Students also emphasised that they wouldn’t feel comfortable giving feedback if the academic co-examiner were not beside them. This appeared to suggest that students would be reticent to give feedback if it were not within a structured learning situation such as the formative clinical assessment.

A significant number of students [57% (51/89)], found that having to make a judgement and provide feedback, which necessitated defining and articulating others’ strengths and weaknesses, made them reflect and critically evaluate their own knowledge, skills, and how they would have presented the same case.

So I learnt, from articulating the things that they did or didn’t do very well or missed out, I learnt just as much from that as they did. S31

Students found that it helped them to understand the priorities of presenting, and also identified weaknesses in their own knowledge.

You compare that to yourself …and well, I wouldn’t have done that much better, so I really need to practice in this area. S2

Seventeen percent (15/89) of students placed value on the professional experience and practice they gained from giving feedback to their peers, stating that it is a “core skill” that “must be learnt”.

It just gives you some exposure to how you should be doing it, like do you think you did well, what do you think you could do better… I think it’s good to have for the future. S17

## Discussion

We specifically sought to investigate students’ perceptions of their ability to provide feedback to their peers, and the challenges and benefits that the experience brought.

While students generally felt confident to provide feedback to their peers, they were apprehensive about providing negative feedback, and concerned about the quality of their feedback, with a number of students indicating further training would be beneficial. Despite student concerns, the majority (90%) found providing feedback to be a useful learning activity. Students perceived the benefits to include development in their understanding of knowledge content and development of required skills in professionalism. They also valued the opportunity to contribute to the education of others.

The main area of concern raised by students was with providing negative feedback to peers and the associated social discomfort. Although most students reported feeling comfortable giving positive feedback to their peers, a significant number of students reported that they were uncomfortable giving negative feedback. Our focus group findings suggest possible explanations that are well supported by current literature. Students had concerns over the influence that negative feedback may have on friendships, with many of the students having established relationships with their colleagues [15]. However, students found the positive critique method helpful, as it provided a standard, structured framework that all students were expected to follow; and it provided an opportunity for the student examinee to lead into the negative aspects of their performance [5].

Students also expressed concerns about the accuracy of their feedback and the adequacy of the training received. As reported elsewhere [7,16], students were worried about their own knowledge, skill and ability to provide appropriate feedback to their peers. A large proportion of students in this study indicated that they needed further training in the provision of constructive feedback to peers. They felt that further training would improve their competence and confidence in articulating accurate and useful feedback. It is thought that with adequate training by faculty, the act of giving feedback to their peers can help to provide an effective learning experience for students and improve the accuracy of feedback [3,17,18]. Within the one hour of training in preparation for the long case assessment, approximately 10 minutes was devoted to describing the positive critique method. It is possible we underestimated student feedback training needs and the apprehensions they might have about providing negative feedback. Greater explanation and opportunity to practice the positive critique method may benefit them.

Although students expressed concerns about their ability to provide feedback, and faced particular challenges, the learning experience was highly regarded. Qualitative responses from students indicate that they felt providing feedback to peers helped to inform self-assessment and review their own knowledge and clinical skills. By being required to give feedback, students are driven to engage, analyse and verbalise what they know, and to realise and address their own knowledge gaps [3]. Literature suggests that preparing, assessing, and providing feedback to peers offer cognitive benefits that are different to simply learning subject content [19]. The scaffolding that is used by students to provide feedback to peers helps students to learn more deeply. Further to this, medical graduates are expected to be skilled in lifelong learning and it has been suggested that experience in peer feedback helps students to gain competence in reflecting on and expanding their own knowledge [20,21].

Although peer review is a common requirement amongst medical staff, it is rarely formally taught in medical schools [22,23]. Our students expressed appreciation for the teaching and practice in feedback skills that was afforded to them, regarding the activity as an important educational tool in developing professionalism attributes [24,25]. The ability to give feedback is said to improve employability skills, such as communications skills, problem solving, decision making and responsibility [7,16].

### Limitations

This study only considers students’ perceptions, and does not provide data on any objective observed or measured ability of students to provide peer feedback.

## Conclusion

While students have concerns about providing feedback to peers, they were able to recognise educational benefits to the process, including knowledge and skills development, and the development of professionalism attributes. They found the positive critique method useful, as it allowed them to deliver feedback in a standard and professional manner. However, further training and explanation in the delivery of feedback may enhance students’ learning experience in the formative long case examinations and better prepare students to enter the medical workforce.

## Competing interests

The authors declare that they have no competing interests.

## Authors’ contributions

AB: study concept and design, analysis and interpretation of data, drafting of manuscript. CR: analysis and interpretation of data, critical revision of manuscript for important intellectual content. KB: analysis and interpretation of data, critical revision of manuscript for important intellectual content. CM: analysis and interpretation of data, critical revision of manuscript for important intellectual content. All authors read and approved the final manuscript.

## Pre-publication history

The pre-publication history for this paper can be accessed here:

http://www.biomedcentral.com/1472-6920/13/79/prepub
